# Development and Validation of the IC3: An Online Remote Assessment Technology for Deep Phenotyping and Monitoring of Cognitive Impairment After Stroke

**DOI:** 10.1177/10731911251381572

**Published:** 2025-10-28

**Authors:** Dragos-Cristian Gruia, Valentina Giunchiglia, Aoife Coghlan, Sophie Brook, Soma Banerjee, Joseph Kwan, Peter J. Hellyer, Adam Hampshire, Fatemeh Geranmayeh

**Affiliations:** 1 Imperial College London, UK; 2 Imperial College Healthcare NHS Trust, London, UK; 3 Harvard Medical School, Boston, MA, USA; 4 King’s College London, UK

**Keywords:** cognition, stroke, neurology, digital health, neuropsychology, validation, cognitive assessment

## Abstract

Automated cognitive assessments tailored to specific clinical scenarios have the potential to revolutionize health care and clinical research. Stroke survivors experience significant burden from underdiagnosed cognitive deficits. To address this, we developed a digital cognitive battery (IC3 [the Imperial Comprehensive Cognitive Assessment in Cerebrovascular Disease]) highly optimized for stroke survivors, and specifically designed for unsupervised administration in patients with mild to moderate stroke, thus enabling detailed remote diagnosis and monitoring of a variety post-stroke cognitive impairments. In a study involving 90 stroke survivors and over 6,000 age-matched healthy adults, the battery demonstrated high concordance with the Montreal Cognitive Assessment (MoCA), a commonly used supervised clinical neuropsychological assessment (*r* = .58, *p* < .001) and close correlation with patients’ quality of life (*r* = .51, *p* < .001). In patients deemed to be cognitively unimpaired based on the standard MoCA cut-off (≥26/30, education-corrected), IC3 detected prevalence of impairment as high as 54% in a subset of tasks (*M* = 30.2%, range = 4%–54%). Importantly, performance on the IC3 remained consistent in both supervised and unsupervised settings in the controls, with minimal learning effects over time. This work provides the first evidence of the robustness and clinical potential of this technology for remote application in stroke, and potentially other neurological settings.

Stroke is a leading cause of death and disability globally, with cognitive sequelae affecting three-quarters of survivors (Douiri et al., 2013). The spectrum of cognitive impairments associated with stroke encompasses domain-specific deficits, such as aphasia, neglect, and memory impairment, as well as domain-general deficits usually associated with co-existing small vessel disease such as executive/attentional dysfunction and reduction in processing speed (Hamilton et al., 2021). Collectively, these impairments have a detrimental impact on post-stroke recovery, engagement with therapeutic interventions, and lower quality of life among patients (Stolwyk et al., 2021, 2024). Consequently, early detection of these impairments has been recommended by key stake holders and national and international guidelines for stroke management (Hill et al., 2022; McMahon et al., 2022; Party, 2023).

Despite a lack of universally accepted approaches for identifying post-stroke cognitive deficits, there is consensus that stroke survivors should be screened and monitored for cognitive impairment using a stroke-specific cognitive assessment (McMahon et al., 2022; Quinn et al., 2021). There remains considerable variability in clinical practice, with deployed assessments ranging from extensive neuropsychological test batteries tailored to a specific cognitive domain (e.g., language), to global measures of cognition using brief screening tools like the Montreal Cognitive Assessment (MoCA) which were not developed for stroke (Nasreddine et al., 2005) This choice is often driven by personal preferences, availability, cost and time pressures, leaving little prospect for Generalizability of findings across sites.

Availability of cost-effective and scalable screening technology that provides stroke-specific deep phenotyping of cognition would be transformative for clinical diagnosis, as well as enabling much-needed large-scale population-based research for studying the mechanisms of post-stroke cognitive recovery. To address this gap, we present a novel digital adaptive technology: the Imperial Comprehensive Cognitive Assessment in Cerebrovascular Disease (IC3). IC3 is a digital assessment battery highly optimized for stroke survivors and designed to require minimal input from a clinician in detecting both domain-general and domain-specific cognitive deficits in patients after stroke. Its purpose is to provide a comprehensive profile of performance across cognitive domains known to be impaired following stroke, including memory, language, executive function, attention, numeracy, praxis, as well as hand motor ability and clinical and neuropsychiatric questionnaires.

Our overall objective was to develop, evaluate, and validate the IC3 cognitive monitoring technology. To this end, we employed participant cohorts of healthy older adults and stroke survivors, along with a thorough set of statistical analyses, across four separate aims outlined in the following paragraphs.

First, we aimed to obtain extensive normative data derived from >6,000 UK-based older adults using the IC3 technology, highlighting its ability to map cognition at a large scale, in a time- and cost-efficient manner. Leveraging this large sample, we were able to account for the effects of demographic and neuropsychiatric variables as well as language proficiency, dyslexia, and device on cognitive performance. These were subsequently used to create patient-specific predictive scores based on novel state-of-the-art Bayesian regression modeling.

Second, we assessed IC3’s validity as a remote cognitive screening tool through a robust set of sub-analyses in controls that aimed to (a) quantify its reliability and feasibility, (b) internal consistency, (c) equivalence in performance between supervised and non-supervised settings, and (d) learning effects across four timepoints.

Third, we aimed to examine whether the IC3-derived patient-predictive scores adequately captured group differences in cognitive performance between patients with stroke and demographically matched healthy adults. Further, we assessed its psychometric validity by conducting a factor analysis in patients to examine whether IC3-derived scores map intuitively onto cognitive domains typically affected by stroke.

Fourth, we aimed to demonstrate that IC3-derived patient scores map onto well-established first-line clinical screening tools (cf. MoCA) and patient-reported functional outcomes (i.e., quality of life). We assessed the sensitivity of the IC3 technology in detecting cognitive impairments against the MoCA, and tested whether the IC3 is able to capture impairments not detected by the MoCA. We discuss the results in relation to the feasibility and validity of the IC3 battery as a technology for monitoring cognition across populations with stroke and cerebrovascular diseases.

## Method

### Participants

The different participant cohorts employed in this study are described below and shown in Figure 1.

**Figure 1.**
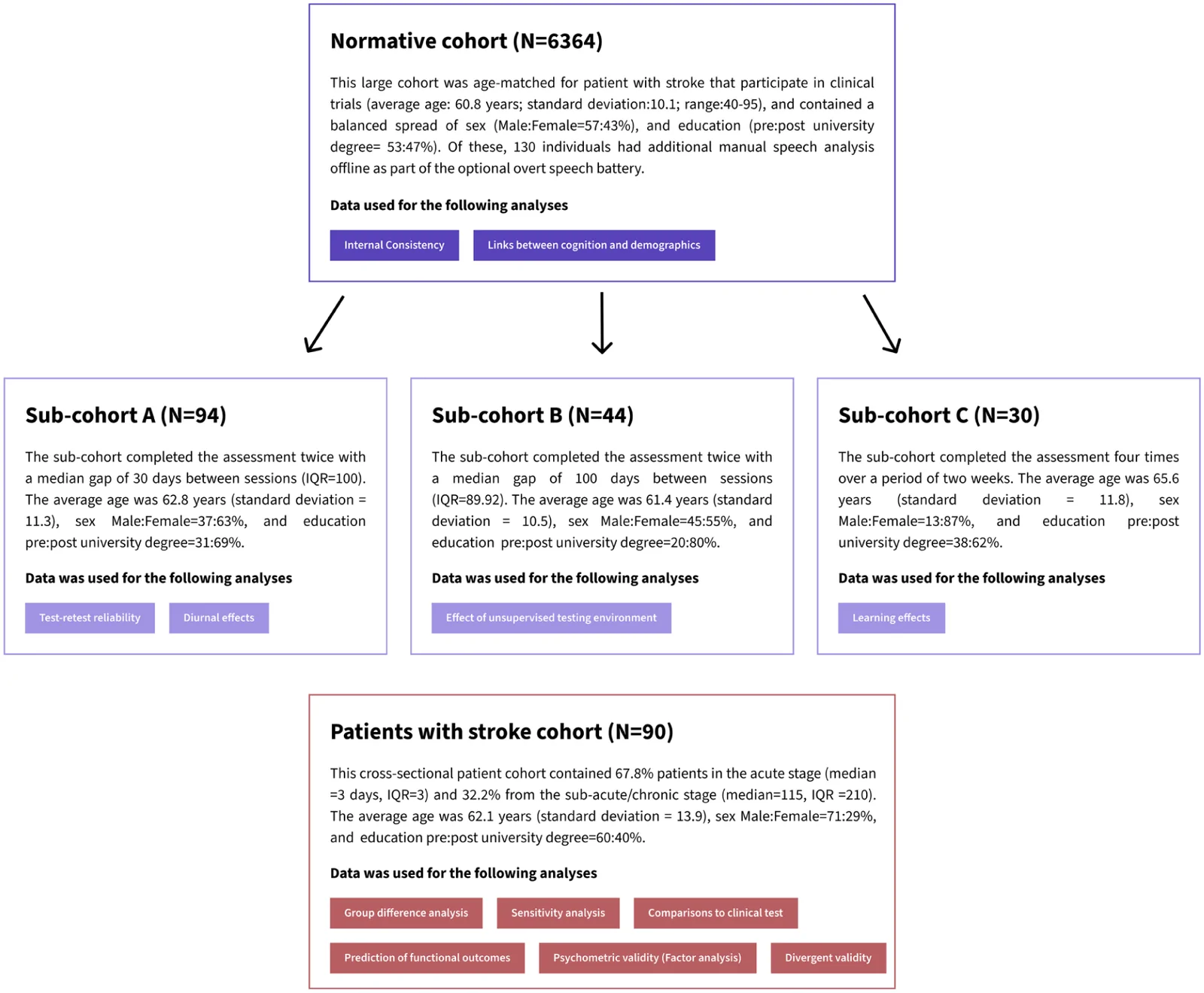
Overview of the Study Cohorts Discussed in This Paper, Along With Their Corresponding Analyses (Colored Boxes). A large normative sample (*N* = ~6K older adults) was used to examine the links between cognitive performance and confounding variables, its psychometric validity, and internal consistency (dark purple). Three sub-cohorts of this normative data underwent further cognitive assessments to determine test–retest-reliability, diurnal effects, environmental effects, and learning effects (light purple). A smaller group of patients with stroke were additionally tested to assess the feasibility and validity of using IC3 in both the acute and chronic phase of stroke recovery (maroon).

#### Normative Cohort (Figure 1, Aim 1)

A study invitation was extended to 25,000 individuals, over the age of 40, residing in Great Britain, all of whom had previously participated in the Great British Intelligence Test (a nationwide initiative aimed at mapping cognition within the general population) and consented to being recontacted for other studies (Hampshire, 2020). Ultimately, 7,095 healthy older adults provided their consent and completed parts of the IC3 cognitive battery, with 5,639 participants (79.5%) successfully completing all 22 tasks. The data were collected remotely online, between October and November 2022. In addition, we collected data from 138 individuals via the Imperial Clinical Research Facility participant registry for reliability and validity purposes. The total sample comprised 7,233 healthy individuals, of whom 6,364 were retained in the normative cohort following participant exclusion and data pre-processing procedures. Participation in the study was voluntary with no monetary incentive.

#### Normative Sub-Cohorts Used for the Reliability and Validity Analyses (Figure 1, Sub-Cohort A–C, Aim 2)

A smaller sample of controls who performed the assessment multiple times was collected separately to test the battery’s reliability and validity. These were recruited using the Imperial Clinical Research Facility participant registry. A total of 94 participants (Sub-cohort A) completed the assessment twice, as part of the test–retest reliability analysis. Of the 94 participants, 44 (Sub-cohort B) performed the assessment both supervised (in person), and unsupervised (remote) with the order counterbalanced. A separate subset of participants (*N* = 30; Sub-cohort C), distinct from those previously described, completed the assessment remotely on four occasions over a 2-week period to enable the evaluation of potential learning effects. No monetary reward was provided, aside from travel reimbursements where appropriate.

#### Patients With Stroke Cohort (Figure 1, Aims 3–4)

Patients with radiologically confirmed stroke were recruited from the Imperial College Healthcare NHS Trust. Exclusion criteria included pre-stroke diagnosis of dementia, pure brain stem stroke, severe visuo-spatial problems, severe mental health diagnoses, fatigue limiting engagement with the IC3 beyond 15 min, and inability to understand task instructions. Consecutively recruited patients underwent the digital IC3 assessment (see Table 1 for detailed demographic information). The scores were compared with clinical pen-and-paper cognitive screens (MoCA). The MoCA scores presented in this paper are corrected for education, by adding one point to the total score of individuals with ≤12 years of education.

**Table 1.** Demographic Characteristics of the Normative (N = 6,364) and Stroke (N = 90) Samples, as Described in Figure 1.

Characterstics	Neurologically healthy sample (*N* = 6,364)	Stroke survivors sample (*N* = 90)
* Age in years (Mean ± *SD*; Range)	60.8 ± 10.1; 40–95	62.1 ± 13.9; 26–90
* Biological sex (%Male: Female)	42.7:57.3	71.1: 28.9
* Education %:		
Secondary school ≤16 years (≤GCSE-educated)	15.5 (*N* = 984)	12.2 (*N* = 11)
Secondary school, aged 18 years (A-levels educated)	37.2 (*N* = 2,364)	47.8 (*N* = 43)
Bachelor’s degree	28.8 (*N* = 1,835)	20.0 (*N* = 18)
Postgraduate Degree	18.5 (*N* = 1,181)	20.0 (*N* = 18)
* Device used %:		
Smart Phone	20.5 (*N* = 1,307)	4.4 (*N* = 4)
Tablet	20.8 (*N* = 1,325)	87.8 (*N* = 79)
Computer	58.7 (*N* = 3,732)	7.8 (*N* = 7)
* With English as second language %:	3.6 (*N* = 231)	33.7 (*N* = 31)
* ^†^ % with Anxiety Diagnosis	5.4 (*N* = 343)	1.1 (*N* = 1)
* ^†^ % with Depression Diagnosis	7.3 (*N* = 466)	3.3 (*N* = 3)
* ^†^ % with Dyslexia Diagnosis	4.0 (*N* = 255)	0.0 (*N* = 0)
**% with Cerebrovascular disease risk factors**		
Ischemic heart disease	4.2 (*N* = 270)	13.04 (*N* = 12)
Diabetes High blood pressure	6.6 (*N* = 416) 27.1 (*N* = 1,724)	17.39 (*N* = 16) 42.39 (*N* = 39)
High cholesterol	20.1 (*N* = 1,282)	19.57 (*N* = 18)
Kidney disease	1.4 (*N* = 87)	1.09 (*N* = 1)
Alcohol Dependency	1.2 (*N* = 78)	1.09 (*N* = 1)
Over-weight	29.7 (*N* = 1,889)	13.04 (*N* = 12)
Long-term smoker	3.9 (*N* = 253)	4.35 (*N* = 4)
Ex-smoker	24.5 (*N* = 1,559)	18.48 (*N* = 17)
**Time (min) taken to complete the assessment**		
Full IC3 battery, excluding participant-initiated breaks(Median ± IQR; Range)	38.4 ± 7.5; 25.15–196.94	55.92 ± 27.42; 36.93–187.09
**Time (min) taken to complete the questionnaires**		
(Median ± IQR; Range)	3.01 ± 0.98; 1.36–28.84	4.94 ± 3.42; 1.75–19.59
**Stroke representation**		
**Days since stroke** (Median ± IQR; Range)		5.0 ± 31.0; 0–556
**% Stroke Phase:**		
Acute (0–14 days)		67.8 (*N* = 61)
Subacute (15–180)		18.9 (*N* = 17)
Chronic phase (>180 days)		13.3 (*N* = 12)
**% Stroke Etiology:**		
Ischemic		92.2 (*N* = 83)
Hemorrhagic		7.8 (*N* = 7)
**% Hemisphere affected**		
Left		50.0 (*N* = 45)
Right		40.0 (*N* = 36)
Bilateral		10.87 (*N* = 9)
**MoCA** (Mean ± *SD*; Range)		21.91 ± 5.95; 3–30
**NIHSS** (Mean ± *SD*; Range)		3.03±2.35; 0–9

*Note*. MoCA = Montreal Cognitive Assessment. NIHSS = National Institute of Health Stroke Scale.

† Self-reported diagnosis with concurrent symptoms and receiving anxiolytic/antidepressant medication at the time of the assessment.

* Covariates that were accounted for in the normative sample.

All participants gave informed consent. The data was acquired as part of a longitudinal observational clinical study approved by UK’s Health Research Authority (Registered under NCT05885295; IRAS:299333; REC:21/SW/0124). Patients also underwent blood biomarker testing and brain imaging, which will not be analyzed in this paper. A lesion overlap map shown in Supplementary Material 5.1 demonstrates the lesion distribution in patients who had brain imaging done (MRI).

### Design Features and Development Process of the Novel Stroke-Specific Assessment Technology

To ensure that the cognitive battery is optimized for use with stroke survivors, both the task design and interface were informed by (a)) in-house expertise in cognitive neuroscience, (b) national and international clinical guidelines for stroke (McMahon et al., 2022; NICE, 2023; Quinn et al., 2021), and (c) previously validated pen-and-paper stroke-specific assessments of cognition (Bickerton et al., 2015; Demeyere et al., 2015, 2021; Swinburn et al., 2004). Specifically, tests to detect predictable domain-specific impairments commonly seen after stroke syndromes, but rare in diffuse neurodegenerative conditions, were included (e.g., neglect, dyscalculia, aphasia, apraxia assessments). Since neurodegenerative co-pathologies are frequent in patients with stroke (Pendlebury & Rothwell, 2019), we also incorporated more domain-general assessments such as memory, executive function, and attention. Accordingly, the language and calculation tests were inspired by the Comprehensive Aphasia Test (Swinburn et al., 2004); neglect, praxis, and executive-function tests by the Birmingham Cognitive Screen (Bickerton et al., 2015) and Oxford Cognitive Screen (Demeyere et al., 2015); and memory and attention tasks drew from the Addenbrooke’s Cognitive Examination (Mioshi et al., 2006), to develop new digital tests and stimuli suitable for remote unsupervised assessments. Several tests of processing speed (based on reaction time [RT]) were included to capture subcortical small vessel disease co-pathology. Additional tests available through the Cognitron platform (Hampshire et al., 2024), assessing executive function, attention, and hand motor function, were adapted and included in the battery. Finally, established neuropsychiatric questionnaires relevant to stroke and key demographics were also collected. These decisions shaped the initial task design and development of the cognitive battery. A summary of all design decisions can be found in Figure 2.

**Figure 2.**
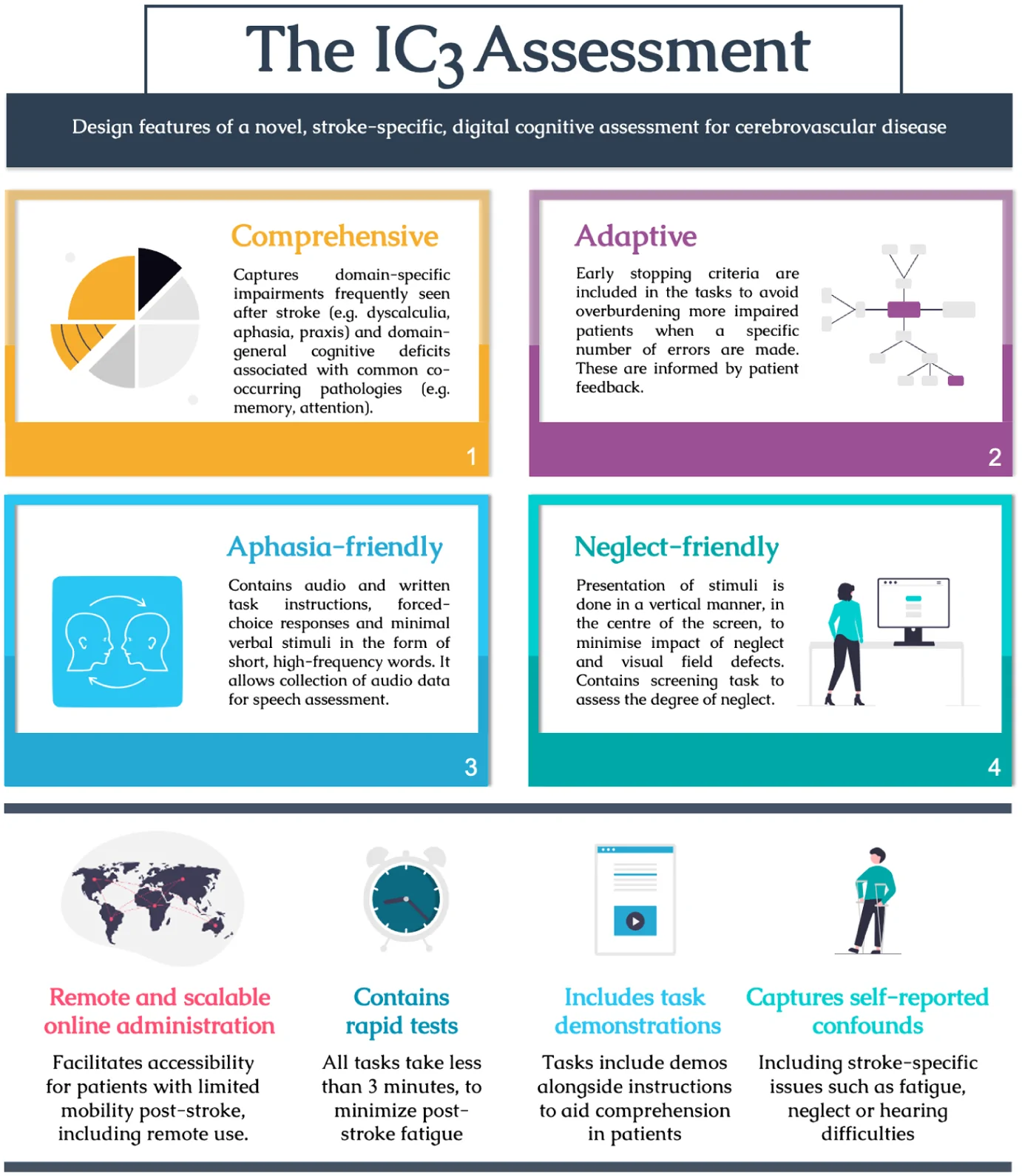
Infographic Summarizing the Core Design Features of the IC3 Digital Cognitive Assessment Optimized for Stroke-Specific Applicability.

A key design consideration was the ability of the test to be self-administered by the patient ± carer support in the absence of a clinician. All tests were designed to be completed using just one hand—whether dominant or nondominant—to minimize the impact of common upper limb impairment post-stroke. The tests were made as inclusive as possible with respect to level of language function when language was not the primary focus. This was achieved through the use of simple, high-frequency words and forced-choice multiple-option questions. To minimize the confounding effects of language comprehension and maximize understanding of task instructions, both audio and written instructions were made available in all tasks. Patients had the option to repeat the instructions without limits and complex tasks had practice trials to ensure understanding. In addition, short demonstration videos accompanied the instructions to enhance task comprehension. Tasks that did not primarily assess language employed non-verbal stimuli. Within language domain, an optional feature was introduced to capture overt speech—dependent on patient’s permission to access the device’s microphone—so that speech impairments could be directly evaluated.

In addition, the tasks were designed to minimize the confounding effect of neglect, when visuo-spatial ability was not the target domain. This was achieved by aligning the stimuli centrally on the screen where possible, to minimize the requirement for visuo-spatial attention on the extremes of the left and right of the screen. An in-built neglect and gross visual acuity screening task (Pear Cancelation) allowed visuo-spatial ability to be estimated.

Further design modifications were made following an iterative patient engagement process ensuring that (a) the cognitive tests were intuitive, (b) the instructions were comprehensible, (c) patients could complete the assessment remotely with minimum supervision, and (d) the burden of the assessment was acceptable. Finally, to minimize fatigue, patients with stroke were permitted to take a break in-between tasks at any time, and the length of each test was capped at 3 min.

### Cognitive Tasks, Speech-Based Tasks, and Neuropsychiatric Questionnaires

A graphical overview and a detailed description of the cognitive assessments are available in Figure 3 and Supplementary Material 1, respectively. These cover 18 short cognitive tasks with 4 additional optional speech production tasks, collectively covering a wide range of cognitive domains known to be affected post-stroke. The tasks were followed by clinically validated questionnaires (Apathy Evaluation Scale Fatigue Scale, Geriatric Depression Scale, and Instrumental Activities of Daily Living; Herrmann et al., 1996; Krupp et al., 1989; Lawton & Brody, 1969; Marin et al., 1991).

**Figure 3.**
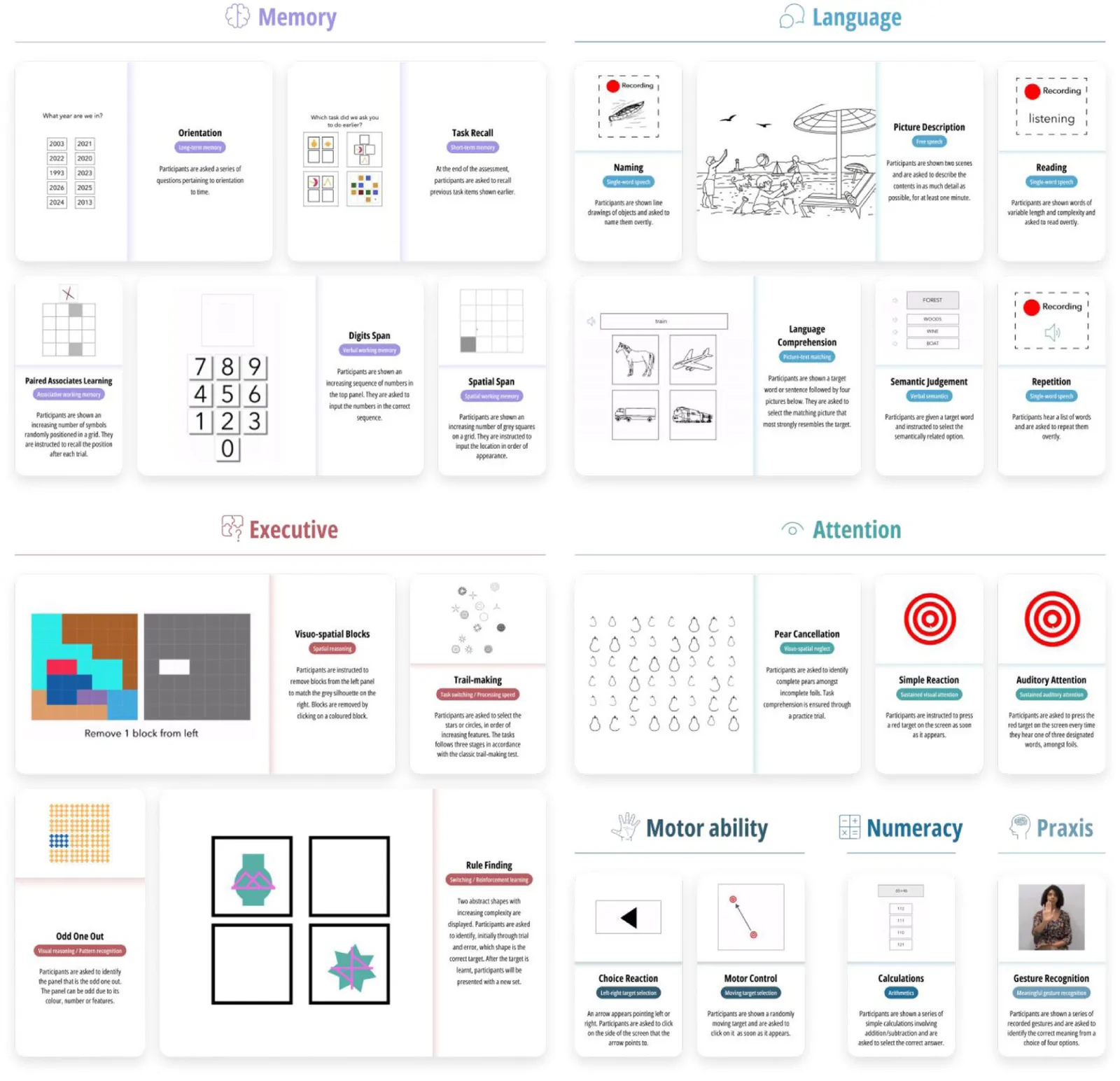
Graphical Overview of 22 IC3 Tasks Organized by the Main Cognitive Domains Tested: Memory, Language, Executive, Attention, Motor Ability, Numeracy, and Praxis. The four optional speech production tasks (naming, repetition, reading, picture description) allow speech to be recorded and manually analyzed offline.

#### Primary Outcomes

Primary outcome variables were based on accuracy rather than speed in most tasks, to avoid the confounding effects of impaired motor reaction times commonly observed after stroke. One exception to this rule is the simple reaction time (SRT) task, which inherently relies on time-based measures to assess processing speed and attention. For the optional speech tasks, the performance was assessed verbatim and offline, by a speech and language therapist, using accuracy-based metrics (e.g., number of correctly spoken words). Detailed task-level information pertaining to the task-specific primary and secondary outcomes are presented in Supplementary Materials 1–2.

IC3 is deployable via a web-browser on practically any modern smartphone, tablet, or computer/laptop device via a weblink and is implemented through Cognitron (https://www.cognitron.co.uk/), a state-of-the art platform for remote neuropsychological testing that is rapidly being adopted by large-scale population studies both in the UK and internationally (Del Giovane et al., 2023; Hampshire et al., 2024).

### Cognitive and Speech Data Pre-Processing

#### Normative Cohort Pre-Processing

Given the remote nature of the cognitive testing, in order to ensure that the normative data was derived from fully engaged healthy participants who understood the task instructions, we implemented three levels of data filtering. These stages include filtering at subject-level, task-level, and trial-level as described below. Distributions of cleaned cognitive task scores can be found in Supplementary Material 5.11.

#### Subject-Level Data Filtering

Exclusion criteria at participant level was based on self-reported (see Supplementary Methods) presence of:

1. Neurological co-morbidities (*N* = 407).
2. Family history of young-onset (<60 years) dementia (*N* = 91).
3. Lack of engagement in the tasks (*N* = 16).

An additional exclusion criterion was failure on two simple cognitive screens (orientation to time: <2/4 correct and pear cancelation: <80%; *N* = 6).

#### Task-Level Data Filtering

The distributions of the primary and secondary outcome measures for each of the 22 tasks were examined to identify outlier performance. Considering the short duration of each task (~2–3 min), and the unsupervised context of assessment, individual tasks were excluded for each participant based on the following time- and performance-based heuristic criteria.

1. Changing browser tabs or minimizing the assessment browser ≥3 times, or for >10 s at a time, during each task.
2. >1 min keyboard inactivity during each task (suggestive of lack of engagement).
3. Failure on practice trials on any given task.
4. Outlier performance, defined as control group mean ± 3 standard deviations (*SDs*). The exclusion criteria were less punitive for tasks with low variability and ceiling effect; for instance, in the Orientation task (where 1 point is acquired for correct orientation to time, day, month, and year), using this approach would have excluded any participants with less than the maximum possible score. Thus, a less stringent criteria of a score <2 (%50 correct) was adopted for this task.
5. Failure to make a response on >50% of the trials within the allocated task duration.
6. For the optional speech production tasks requiring manual annotations (see Supplementary Results 1.2.), speech tasks from controls (*N* = 1) with pathological speech were excluded.

The amount of data removed through this process at the task-level was an average of 2.2% (range across all tasks: 0.05%–6%).

#### Trial-Level Data Filtering

The trial-by-trial variability in performance within each task was examined for signs of non-engagement. This approach was task-dependent, as non-engagement manifested differently depending on the rules of the task:

1. Where the primary outcome measure of a task was response time–based (e.g., SRT), trials with RT < 200 ms were excluded, as these responses were likely to reflect premature or repeated responses independent of the stimuli. Tasks where >50% of trials fulfilled this criterion, coupled with unusually low accuracy (defined as <2 *SD* below group mean performance) were excluded.
2. Trials with excessively prolonged RTs were excluded when determining RT (defined as >3 *SD* above the mean RT of the normative sample for that task), as a sign of technical errors or non-engagement.
3. For the optional speech production tasks requiring manual annotations, trials were excluded if there were technical or quality sound recording problems (see Supplementary Results 2.5).

Trial-level exclusion was applied only to RT metrics. In this study, the only task using RT as part of its primary outcome measure was the SRT, for which 1% of trials were excluded.

#### Patient Cohort Pre-Processing

During the self-administration of the tasks, a trained researcher would flag any instances where any technical issues occurred (e.g., task did not load due to loss of internet connection) or the patient did not fully engage in the task (e.g., patient was interrupted part-way through the task by clinical staff). This information was used to exclude specific tasks from further analysis. From the 90 patients that took part in the current study, 3.71% of task-level data was removed according to the above-mentioned exclusion criteria.

### Bayesian Modeling on the Large Normative Sample Derives Patient-Specific Predictive Scores

Constrained by relatively small normative sample sizes, existing cognitive batteries have traditionally been limited to accounting for the effects of demographic factors by stratifying the normative sample into even smaller sub-groups, often limited to one or two variables (age and occasionally education). In this study, we leverage the large normative sample of 6,364 individuals and Bayesian modeling (see below), to create patient-specific predictive scores with higher precision, accounting for eight additional confounding factors (age, sex, education, language proficiency, testing device, depression, dyslexia, anxiety; highlighted with an asterisk in Table 1).

#### State -of-the-Art Bayesian Posterior Predictions for Modeling the Relationship Between Cognition and Confounding Factors in Controls

Bayesian regression analyses containing all eight covariates were performed separately for all tasks, to estimate the effects of each of the covariates on individual task performance. For the optional speech production tasks (repetition, naming, reading), the smaller sample (*N* = 130) precluded the inclusion of depression, anxiety and dyslexia as covariates. (For full details of Bayesian regression models and how the coefficient were derived, see Supplementary Materials 4.1–4.2)

#### Patient-Specific Impairment Thresholds

Bayesian modeling was used to create patient-specific impairment thresholds correcting for the aforementioned confounding variables. This was done by (a) training the Bayesian regression models on the normative sample as described above, (b) using the derived posterior distributions to estimate patient-specific predicted performance, converted to *SD* units, and (c) subtracting the observed patient performance (also in *SD* units) from the predicted performance derived from step “b.” A resulting negative “deviation from norm” score suggests that the patient had a deficit in that specific task by a given magnitude in *SD* units, such that a score of –1 represents an impairment of 1 *SD* from a corresponding demographically matched control group. Using these estimates, boundaries for the severity of the cognitive impairments were arbitrarily assigned as –1.5 (mild), –2.0 (moderate), and –2.5 (severe) *SD* below the mean, in line with previous post-stroke cognitive tests (Demeyere et al., 2021).

## Results

### Normative Sample

#### Participant Characteristics

The cleaned normative sample consisted of 6,364 individuals. The number of participants were varied for each task (*N* = 4,782–6,290) depending on task-specific data filtering and the fact that tasks at the end of the assessment had fewer timepoints. Table 1 outlines the demographics and additional confounding factors that may affect cognitive performance.

#### Relationships Between Cognition and Confounding Demographic Factors in the Normative Sample (Figure 1, Normative Cohort)

Standardized coefficients were obtained from task-specific Bayesian regression models for the eight confounding covariates (mean *R*^2^:11.29%; range: 1.3–53.5). The lower range of the *R*^2^ was driven by tasks with ceiling effects and low inter-subject variability in the controls (e.g., 3.9/4.0 and 3.8/4.00 for Orientation and Task Recall mean performance, respectively).

The strength of the association between each of the eight covariates and cognition are shown in Figure 4 where warm and cool colors represent positive and negative standardized coefficients. Cognitive performance generally worsened with age as shown by negative coefficients shown in purple. The exception is Semantic Judgment in keeping with previous literature demonstrating age-related improvement in language function (Hartshorne & Germine, 2015). Dyslexia and English as a second language had a strong negative effect on performance, particularly on tasks involving language and numeracy skills. Device was a strong confounding factor in tasks that relied on speed and motor dexterity as the main outcome measure (e.g., SRT, Choice Reaction Time, and Motor Control). This association is understandable given faster responses on touch screen compared to mouse/trackpad-operated devices. Higher education levels were related to better cognitive performance across tasks that involved language and numeracy, with the least effect on tasks that primarily captured motor dexterity (e.g., Motor Control, Choice Reaction Task, SRT tasks). Overall, the regression models provide intuitive and interpretable relationships between cognition and the eight confounding factors.

**Figure 4.**
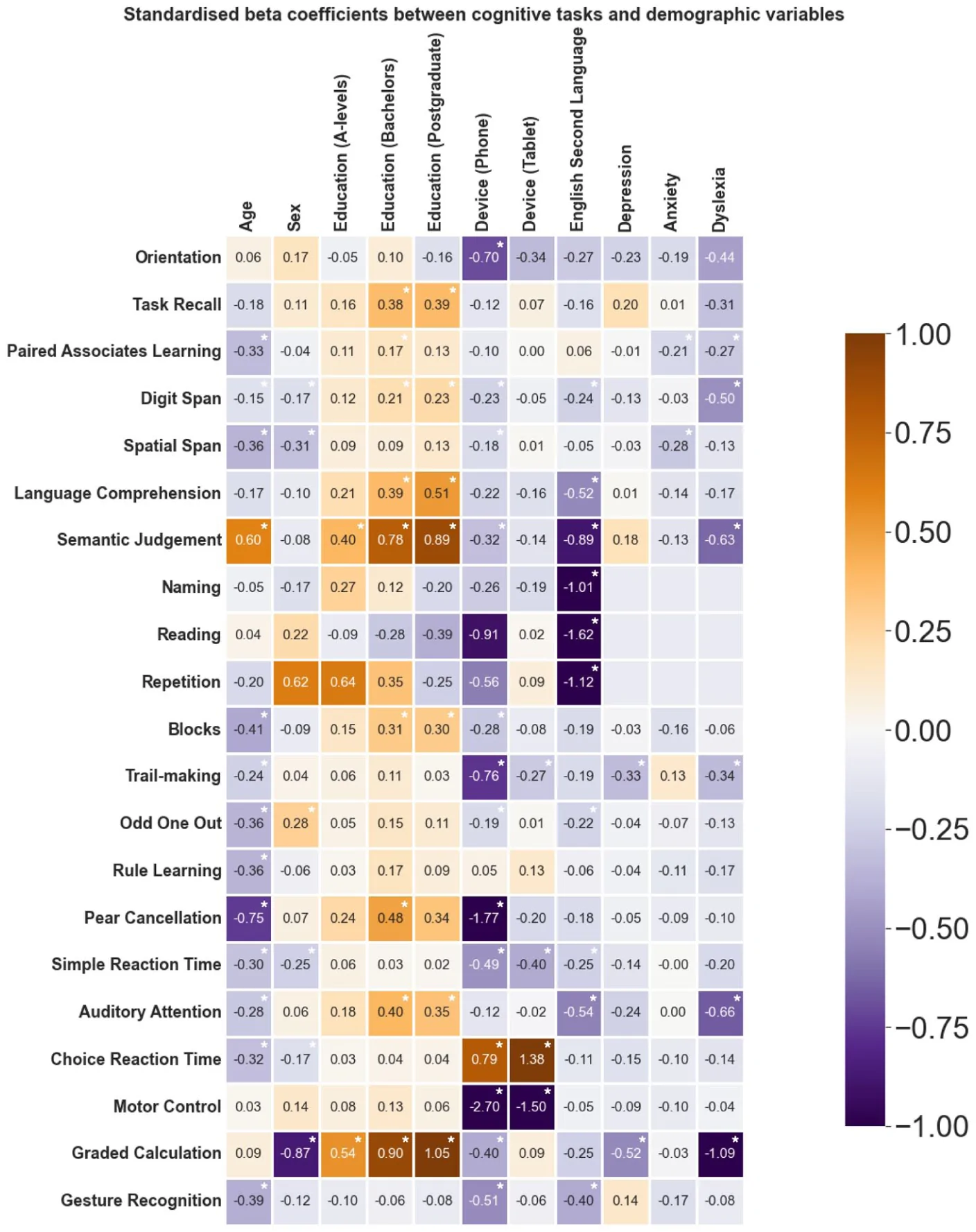
Relationship Between Cognitive Performance and Eight Confounding Factors in the Large Normative Sample, Quantified via Standardized Regression Coefficients. The regression reference categories for Education and Device were “≤GCSE-educated” and “Desktop Computer,” respectively. Negative coefficients (cool colors) indicate the covariate was associated with worse cognitive performance. Empty cells represent coefficients not used in the modeling for the optional speech production tasks, given the smaller normative sample. Regression coefficients for tasks with normally distributed data are in standard deviation units, and those with non-Gaussian distribution are in log-odd units. Positive coefficients are indicative of better performance. “*” = Uncorrected statistically significant predictors.

### Reliability of IC3 Technology

#### Internal Consistency Among Tasks

The participants included in this analysis correspond to the normative cohort (see Figure 1, dark purple). The task-level internal consistency was generally good, particularly for tasks with high variability on the primary outcome measure, with 17/21 tasks showing split-half alpha values surpassing the standard threshold for good internal consistency (α = 0.70; see Supplementary Material 5.3 for a more detailed methodology). Nevertheless, a small subset of tasks exhibited lower alpha values (i.e., Orientation, Task Recall, Gesture Recognition, and Calculation, marked with “^†^” in Table 2). A well-known limitation of the split-half reliability is its dependency on a large number of trials, with shorter tasks having inherently low reliability estimates (Pronk et al., 2022). Thus, the low alpha values may be attributed to the low number of trials (4–6) in these short screening tasks, despite the tasks being preferable in clinical settings due to their lower burden on patients. A further factor is the ceiling effect in these short tasks such that a single error results in a significant change in the trial’s relative ranking and estimated covariance between trials.

**Table 2.** Internal Consistency (Full Normative Cohort) and Test–Retest Reliability (Sub-Cohort A) Analyses of Older Adult Controls.

Domain	Task	Internal consistency		Test-retest reliability sub-study							
		*N*	Alpha	*N*	Mean time 1	Mean time 2	Group mean difference *p*-value	Effect size	Test of equivalence *p*-value	*R*-value	Normally distributed
Memory	Orientation	6,290	0.14^†^	92	3.88	3.9	0.74	0.07	<0.001	0.1^†^	No
	Task Recall	4,782	0.04^†^	91	3.79	3.87	0.34	0.2	<0.001	0.31^†^	No
	Paired Associates Learning	5,721	0.73	87	4.68	4.97	0.29	0.22	0.03	0.31	Yes
	Digits Span	5,279	0.73	86	6.69	7.0	0.1	0.24	0.03	0.59	Yes
	Spatial Span	5,867	0.70	90	5.08	5.22	0.46	0.13	0.01	0.45	Yes
Language	Comprehension	5,025	0.76	91	18.44	18.52	0.49	0.05	0.01	0.45	No
	Semantic Judgment	4,995	0.94	92	22.4	22.58	0.46	0.14	<0.001	0.62	No
	Naming	132ˆ	0.79	85	0.97	0.98	0.41	0.13	<0.001	0.46	No
	Reading	132ˆ	0.72	87	0.99	0.99	0.19	0.23	<0.001	0.12^†^	No
	Repetition	132ˆ	0.83	86	0.99	0.98	0.12	0.33	<0.001	–0.05^†^	No
Executive	Blocks	5,039	0.70	54^‡^	6.52	7.28	0.1	0.22	0.03	0.77	Yes
	Trail-making*	5,189	0.81	82	1.02	1.05	0.92	0.01	<0.001	0.08^†^	No
	Odd One out	5,672	0.75	91	10.73	11.23	0.1	0.23	0.03	0.59	Yes
	Rule Learning	5,485	0.83	85	6.75	7.24	0.1	0.27	0.04	0.42	No
Attention	Pear Cancelation	6,219	0.88	85	0.98	0.99	0.1	0.41	<0.001	0.3^†^	No
	Simple Reaction Time*	5,510	0.95	91	390.66	386.12	0.66	0.06	<0.001	0.68	Yes
	Auditory Attention	5,757	0.73	91	35.24	35.44	0.35	0.15	<0.001	0.32^†^	No
Motor Ability	Choice Reaction Time	5,181	0.88	87	54.59	53.9	0.65	0.08	0.01	0.28	No
	Motor Control	5,208	0.88	93	28.96	28.95	0.97	0.01	<0.001	0.14^†^	No
Numeracy	Calculation	5,681	0.33^†^	91	7.86	7.88	0.82	0.04	<0.001	0.09^†^	No
Praxis	Gesture Recognition	5,280	0.05^†^	91	7.48	7.55	0.59	0.09	<0.001	0.36^†^	No

*Note*. “*N*” = Sample size. “ˆ” = Lower sample size due to tasks requiring manual transcription of overt speech. “Alpha” = 5,000 iterations of permutation-based randomly split-half sampled trial-level data were used to calculate internal consistency. “^†^” = Unreliable alpha values due to lack of variance in controls and low number of trials. Nevertheless, test–retest reliability suggests that all these tasks are stable across time. “Group difference *p-v*alue” = Obtained from a paired-samples *t*-test or Wilcoxon test depending on the stated distribution. “Effect size” = Cohen’s *d*. “Test of equivalence *p*-value” = *p*-value obtained from a parametric on non-parametric test of equivalence dependent on the stated distribution; significant *p*-values equate equivalence. The *p*-values are FDR-corrected. “*R*-value” = Coefficient obtained from a Pearson or Spearman correlation dependent on the stated distribution. “*” = Indicates tasks where higher values suggest worse performance. “^‡^” = Lower *N* caused by exclusion of 32 participants whose version of Blocks differed between sessions due to improvements in task design. “^†^” in *R*-value = Indicates that the correlation values may be low due to lack of variance in both sessions, despite highly similar mean values between sessions.

#### Test–Retest Reliability Across Time

Participants included in this analysis correspond to Sub-cohort A (see Figure 1, light purple). Consistency between sessions was assessed via well-established metrics: (a) group difference analyses, (b) equivalence testing, and (c) correlation analyses. There was no significant difference (FDR-corrected) in performance between two sessions on any of the IC3 tasks with strong equivalence across all measures (see Table 2). There was moderate to high correlation (Akoglu, 2018) between sessions for tasks with high variance within the normative group (*r* = 0.42–0.77 for 9/18 tasks), and smaller correlation for those with low variance due to ceiling effects despite similar group means across sessions (*r* = –0.05–0.36 for 8/18 tasks). Two exceptions to this observed trend were the Paired Associates Learning and Choice Reaction Time tasks which despite having reasonable variance in the normative group, comparable mean performance and test equivalency across the two time points, had relatively low correlation values (*r* = 0.31 and 0.28, respectively). This is explained by the device related variability between the two sessions (31/94 switched devices) affecting performance in Choice Reaction Time, and the expected learning effect in the Paired Associates Learning task. When diurnal associations with performance were examined across inter-session time interval and that of time of day when the assessment was performed, the results showed that these time-related factors did not explain change in cognitive scores across the two sessions (Supplementary Material 5.4). Overall, these results demonstrated a stable performance of the control group on IC3 across time.

#### Remote Monitoring Technology Shows Equivalent Performance Between Unsupervised and Supervised Testing Environment

Participants included in this analysis correspond to Sub-cohort B (see Figure 1). With the exception of two motor tasks (Choice Reaction Time and Motor Control), there were no differences between performance of supervised and unsupervised testing environment (Table 3). Given that the performance on the two motor tasks was strongly dependent on the device type used (Figure 3), additional Bayesian hierarchical regression modeling of these two tasks was conducted with participant ID as the grouping factor, and with device, environment, age, sex, and education, as independent variables. Posterior predictions indicated no effect of environment after accounting for device in either the Choice Reaction Time (uncorrected *p*-value = .36, 95% CI = [–.44,.26]) or the Motor Control Tasks (uncorrected *p*-value = .28, 95% CI = [–.36,.60]). However, we did find a strong effect of device in both tasks (uncorrected *p* = .001, 95% CI = [.33,1.16], and *p* = .0002, 95% CI = [.58,1.87], respectively). This confirmed that the effect of session for these tasks was driven not by the supervised/unsupervised environment but by the device used to perform the assessment. Thus, we conclude that there were no significant direct effects of conducting assessment within an unsupervised testing environment.

**Table 3.** Effects of Supervised versus Unsupervised Testing Environment on Cognition in Sub-Cohort B.

Domain	Task	*N*	*Mean* remote	*Mean* supervised	*p*-Value	Effect size	Test of equivalence	Normally distributed
Memory	Orientation	43	3.977	4.0	1.0	0.22	<0.001	No
	Task Recall	43	3.907	3.907	1.0	0.0	<0.001	No
	Paired Associates Learning	41	4.951	4.878	1.0	0.06	0.01	Yes
	Digits Span	42	7.048	6.762	0.63	0.24	0.03	Yes
	Spatial Span	43	5.116	5.395	0.63	0.27	0.03	Yes
Language	Comprehension	44	18.909	19.045	1.0	0.11	0.02	No
	Semantic Judgment	43	22.744	22.628	0.63	0.14	<0.001	No
	Naming	44	0.98	0.975	0.86	0.11	0.01	No
	Reading	43	0.996	0.996	1.0	0.0	<0.001	No
	Repetition	42	0.987	0.994	0.75	0.26	<0.001	No
Executive	Blocks	39	7.718	7.282	0.88	0.14	0.02	Yes
	Trail-making*	40	0.325	1.475	0.07	0.57	0.03	No
	Odd One out	41	10.927	11.317	0.63	0.21	0.02	Yes
	Rule Learning	40	7.0	7.05	1.0	0.03	0.02	No
Attention	Pear Cancelation	42	0.994	0.993	1.0	0.05	<0.001	No
	Simple Reaction Time*	42	379.605	393.48	0.72	0.19	0.02	Yes
	Auditory Attention	43	35.581	35.512	1.0	0.08	<0.001	No
Motor Ability	Choice Reaction Time	43	51.349	59.395	<**0.001**	1.11	**0.05**	No
	Motor Control	44	29.409	28.091	<**0.001**	0.69	**0.05**	No
Numeracy	Calculation	43	7.907	7.953	1.0	0.13	<0.001	No
Praxis	Gesture Recognition	43	7.721	7.558	0.4	0.31	0.01	No

*Note*. “*N*” = Sample size after pre-processing. “*p*-Value” = Obtained from a paired-samples *t*-test when the sample is normally distributed or from a Wilcoxon test when the sample is not. “Effect size” = Cohen’s *d*. “Test of equivalence” = *p*-value obtained from a parametric test of equivalence when the sample is normally distributed, or from a non-parametric test if the sample is not. Here significant *p*-values >0.05 equate equivalence. All *p*-values are FDR-corrected and shown in bold if significant. “*” = Indicates tasks where higher values suggest worse performance.

#### Online Technology Is Robust to Learning Effects Across Four Timepoints

We examined the potential effect of learning by analyzing performance across repeated testing timepoints in 30 controls (Sub-cohort C) who completed the IC3 four times over the course of 2 weeks. The results showed that IC3 assessment has minimal learning effects within this expected testing time interval (detailed analyses shown in Supplementary Material 5.6).

### IC3 Technology in Patients With Stroke

#### Online Battery Is Sensitive to Group Differences Across all Tasks

Of 601 patients screened acutely for IC3 cognitive testing, 511 were deemed inappropriate for a variety of reasons as shown in Supplementary Results 5.1, Supplementary Table 1. Out of all patients who started the cognitive battery, 94% of patients in our study cohort were able to complete the assessment. With respect to technical difficulties, this was largely evident during the speech capture process for speech production tasks. Specifically, we note that disruptive background noise, caused by the acute clinical setting in which the data was collected, affected the quality of the speech recordings (*N*=12).

Moreover, data from 90 patients with stroke were analyzed (see Figure 1 for cohort information). The IC3 testing was performed during the acute post-stroke phase in 68% (3 ± 3 days [median ± IQR] post stroke) and in the sub-acute/chronic post-stroke phase in 32% (115 ± 210 days post stroke). See Table 1 and Supplementary Materials 5.1.

The task-level average group performance was calculated in “deviation from expected” *SD* units as described above. Healthy controls significantly outperformed patients across all tasks, showing moderate-to-large effect sizes in the majority of tasks after accounting for eight confounding factors (19/21 tasks, *p* < .05 FDR-corrected; *d* >.43 for all tasks). See Figure 5 and Supplementary Material 5.6 for detailed statistical results.

**Figure 5.**
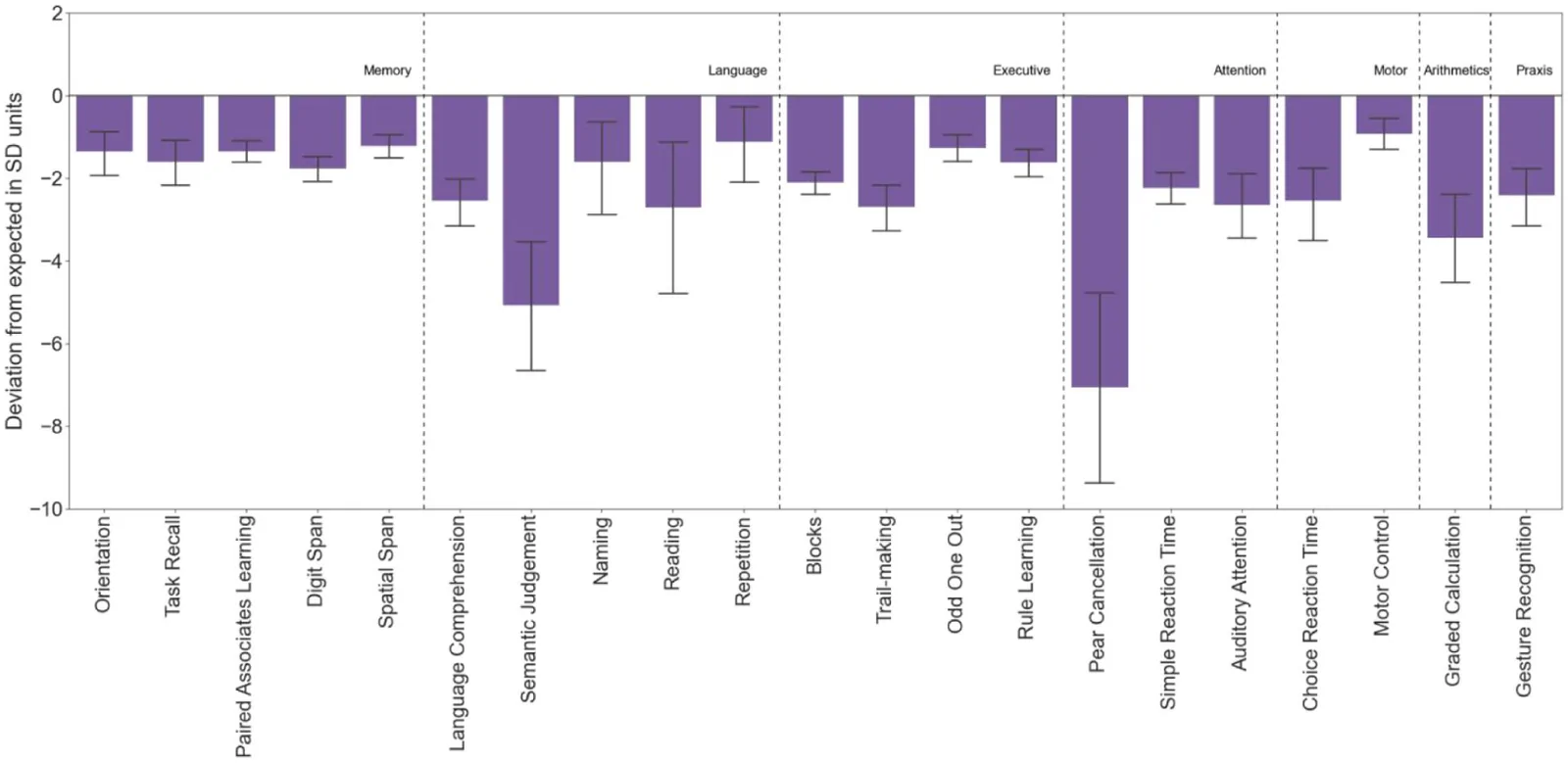
Patients With Stroke Had Significantly Worse Performance Than Controls (FDR-Corrected). Error bar = 95%CI. 0 = mean control performance.

When the data was split by the lesioned hemisphere, those with a right hemisphere lesions no longer showed a group difference for the speech production tasks (naming, repetition and reading) that rely on left hemispheric processing for language function. (Supplementary Material 5.10).

#### Online Battery Correlates With Clinical Neuropsychological Scores and Functional Impairment After Stroke

A data-driven “IC3 global composite score” was derived from factor analysis based on combined data from patients and controls (Supplementary Material 5.7). This had a positive loading on individual tasks and accounted for 47% of the variance (Figure 6A). Six group factors, each loading on a subset of tests were also derived, intuitively mapping onto Executive Function (F1), Language/Numeracy (F2), Working Memory (F3), Attention (F4), Motor Ability (F5) and Memory Recall (F6). The factor analysis fit was robust (CFI = 0.94, RMSEA = 0.01) with good internal consistency (omega = 0.79).

**Figure 6.**
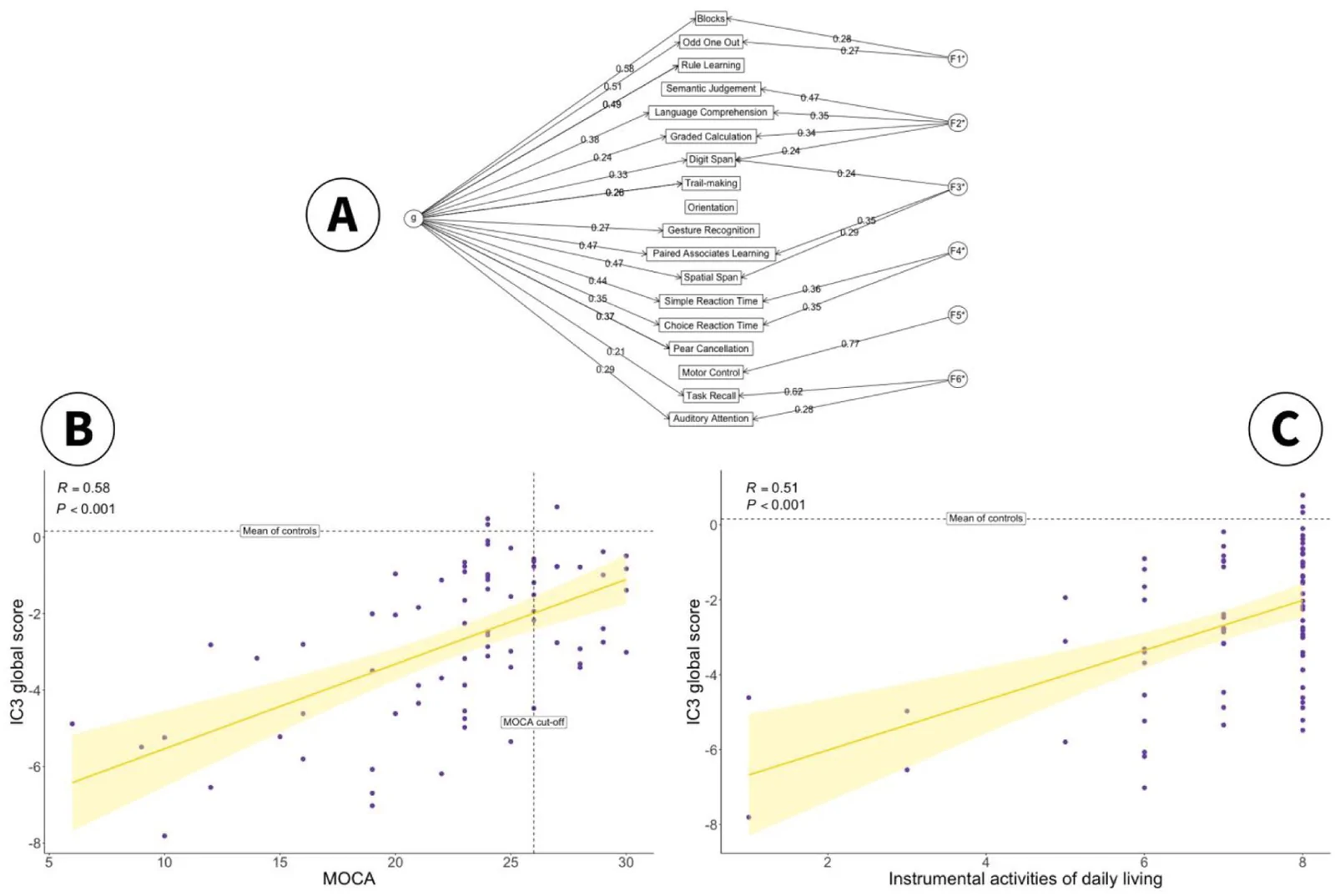
(A) The Solution to a Bifactor Exploratory Factor Analysis on the Combined Dataset of Patients and Controls. The Global Cognitive Measure for the IC3 is Defined as the “g” Factor. The Remaining Six Factors Intuitively Map to Executive Function (F1), Language/Numeracy (F2), Working Memory (F3), Attention (F4), Motor Ability (F5) and Memory Recall (F6). (B) Correlation Between IC3 Global Score (g) and Clinically Validated Total MoCA Score in Patients (N = 80). Vertical Dotted Line: MoCA Cut-Off for Normality. Horizontal Dotted Line: Mean Global IC3 in Controls. Shaded Area Represents 95% Confidence Interval. (C) Relationship Between IC3 Global Score (g) and Post-Stroke Quality of Life Metrics (IADL). Horizontal Dotted Line: Mean Global IC3 in Controls. Error Bar Represents 95% Confidence Interval.

In keeping with task-level results, IC3 global score (*g*) was significantly lower in patients compared to controls (*p* < .001, Supplementary Material 5.7). Furthermore, the IC3 global score and total MoCA scores were significantly correlated in patients, *r*(78) = 0.58, *R*^2^ = 0.33, *p* < .001, Figure 5B, indicating that the IC3 performance maps onto a clinically validated neuropsychological screen.

To assess the external validity of IC3, IC3 global performance was also related to functional impairment after stroke as defined by the Instrumental Activities of Daily Living (IADL) score (Lawton & Brody, 1969). Worse global cognitive performance on the IC3 was associated with worse functional impairment after stroke, *r*(78) = 0.51, *R*^2^ = 0.26, *p* < .001, Figure 5C.

Conversely, MoCA had a considerably weaker relationship with functional deficits post-stroke, explaining approximately half of the variation explained by the IC3, *r*(78) = 0.38, *R*^2^ = 0.14, *p* < .001. A separate linear regression analysis was conducted to quantify this difference, with MoCA and IC3 as independent predictors, and IADL as the dependent variable. The results show that there was no longer a main effect of MoCA on functional impairment when accounting for the IC3 global score (*p* = .22), while the IC3 global score remained highly significant (*p* = .004). Moreover, the inclusion of MoCA only explained an additional 1% of variance, suggesting that MoCA does not bring any additional information beyond what is captured by the IC3.

Furthermore, we demonstrate strong divergent validity for IC3 (Supplementary Material 5.8), as shown by an expected lack of correlation between cognitive performance in patients and variables known not to be related to cognition (i.e., admission cholesterol levels).

#### Online Monitoring Technology Is More Sensitive to Mild Cognitive Impairment Than Standard Clinical Assessment

IC3 showed high sensitivity at both the domain level and the task level (as shown in high true positives in dark purple, Figure 7A). Given that IC3 was specifically designed to detect mild impairment, the sensitivity of the MoCA screening tool was also assessed against the IC3 and found to be weaker (Figure 7A, in yellow). A chi-square test indicated that this difference is statistically significant at *p* < .001. See Supplementary Material 5.9 for details on how the “ground truth” and domains were chosen.

**Figure 7.**
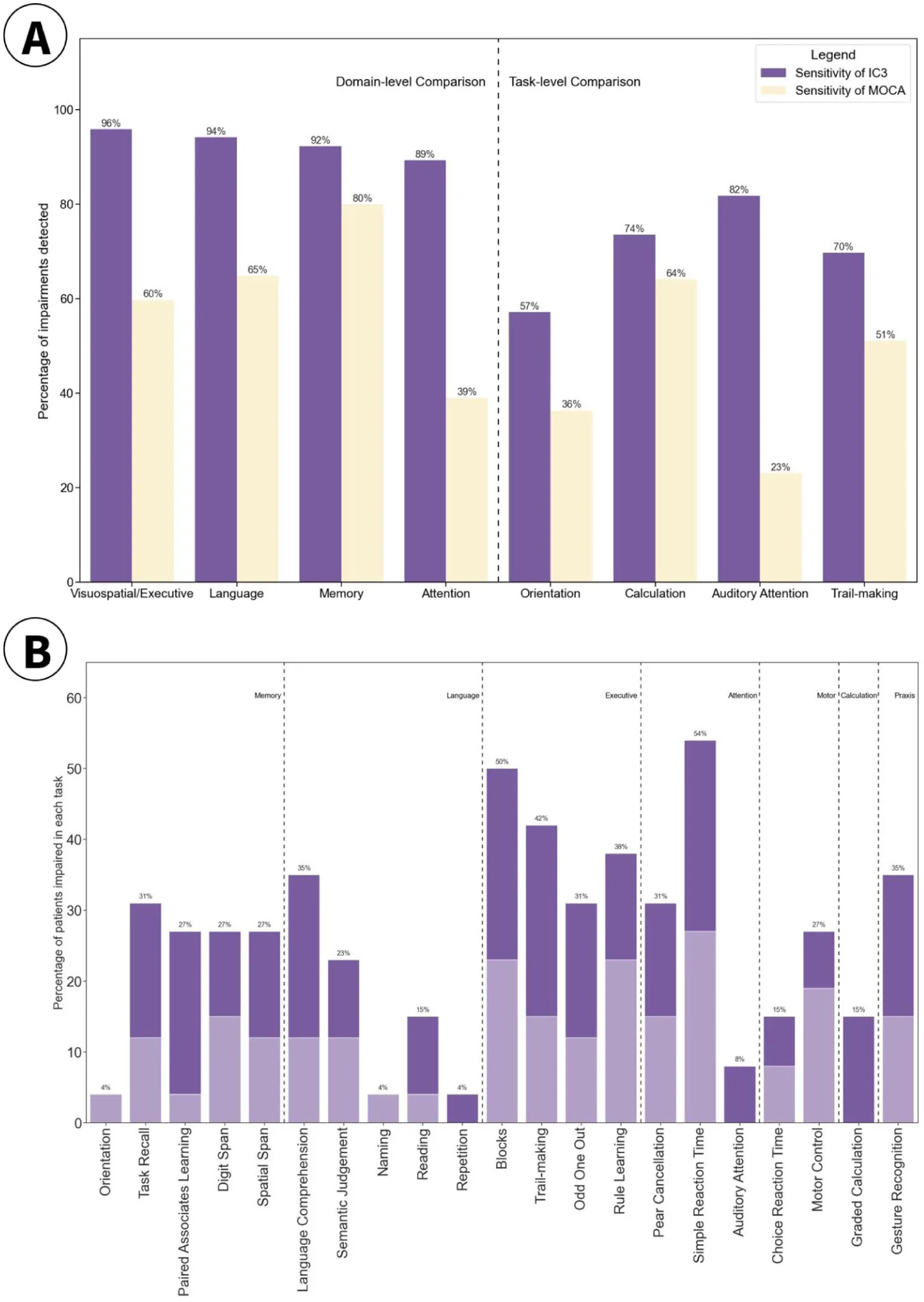
(A) Sensitivity of IC3 Against MoCA (Purple) and vice versa (Yellow) in Detecting Impairment at Domain and Task Level Where a Clear 1:1 Mapping Was Present. (B) Percentage of Patients Classed as Impaired on the IC3, Within a Sub-Group of Stroke Patients That Were Deemed Cognitively Healthy on the MoCA (N = 27). Dark purple = scores from the acute stage of the stroke. Lighter purple = sub-acute/chronic stage.

In addition, as shown in Figure 7B, IC3 was able to detect a substantial proportion of impairment in patients, even when those patients were classed as “healthy” according to their MoCA performance (score ≥26/30). The MoCA scores presented in this paper are corrected for education, by adding one point to the total score of individuals with ≤12 years of education. These impairments were detected in both the acute (dark purple) and the sub-acute/chronic stage (light purple) after stroke, highlighting IC3’s ability to detect mild impairment, undetected by clinical screens, in all stages of recovery after stroke.

## Discussion

The current study evaluated the IC3 battery, a novel digital online cognitive testing technology designed to enable large-scale identification and monitoring of cognitive sequelae after stroke and related vascular disorders. The robustness and reliability of the battery was extensively demonstrated and reinforced through test–retest reliability, equivalent performance between supervised and non-supervised settings, minimal learning effects across multiple timepoints, and high psychometric validity. An extensive normative sample of more than 6,000 older adults from the United Kingdom was systematically gathered and leveraged to calculate patient-specific prediction and impairment scores. While accounting for a wide range of demographic and neuropsychiatric variables, IC3 was able to differentiate healthy controls and stroke survivors across all tasks on the IC3 battery, with effect sizes ranging from moderate to very large (*d* = 0.43–1.59).

Importantly, patient outcomes derived from IC3 demonstrated strong concordance with results from clinical scores available in the patients (MoCA), but with superior sensitivity when detecting mild cognitive impairments and stronger correlation with patient-reported functional impairments post-stroke (IADL). Reassuringly, the convergence validity of the IC3-derived outcomes in patients was balanced against an expected good divergent validity as shown with no discernible correlation with admission cholesterol levels. Collectively, these findings provide compelling evidence supporting the validity of remote digital testing via the IC3 platform as a clinical tool for assessing and monitoring cognition following stroke and related vascular disorders.

Compared to other assessment tools, IC3 battery provides: (a) scalability and cost-effectiveness in post-stroke cognitive monitoring in both health care and research settings, (b) sensitivity to mild cognitive impairments, (c) specificity to deficits found post-stroke, meeting the national guidelines requirements for cognitive assessment in stroke, (d) nuanced response metrics per individual tests (e.g., accuracy, RT, and trial-by-trial variability), and (e) the ability to output automated real-time patient-predictive scores accounting for confounding demographic factors (Giunchiglia et al., 2023; McMahon et al., 2022; Party, 2023; Quinn et al., 2021).

Currently available stand-alone cognitive screening tools that are commonly used in routine clinical care mostly are either not stroke-specific, or not comprehensive. Prominent examples include the MoCA, Mini Mental State Examination, and Addenbrooke’s Cognitive Examination–Revised, which are tailored to detect deficits in neurodegenerative dementias and are not tailored to patients with stroke, who often have domain-specific as well as domain-general deficits (Kurlowicz & Wallace, 1999; Mioshi et al., 2006; Nasreddine et al., 2005). In addition, increasingly used cognitive screens designed specifically for stroke, such as the OCS, although more sensitive than MoCA, are not comprehensive enough to allow a deep cognitive phenotyping and miss the milder end of the severity spectrum (Demeyere et al., 2015). The digital OCS-Plus only assesses memory and executive function and requires a trained staff to administer, thus limiting its scalability and affordability compared to the IC3 (Demeyere et al., 2021). Other digital platforms such as CANTAB (Smith et al., 2013), CogniFIT (Embon-Magal et al., 2022), and Cogniciti (Paterson et al., 2022), largely used/marketed in the setting of neurodegenerative and/or psychiatric disorders, are neither stroke-specific nor capable of providing patient-predictive scores. The IC3 assessment battery developed and applied in this study addresses these shortcomings, building the foundation for routine detailed monitoring of cognition in health care setting, and for scalable large-scale population-based studies of post-stroke cognitive impairment.

A general advantage of digital cognitive testing is its ability to present either different stimuli from a larger pool of standardized available options or randomize the presentation order, hence minimizing the learning effects due to stimuli familiarity and order effects. Indeed, we observed minimal learning effect in all tasks but one, the odd one out. This is in keeping with other reports that identify minimal learning effects in digital cognitive tests (Oliveira et al., 2014).

With respect to the normative cohort, the sample size under discussion (*N*>6,000) is an order of magnitude larger than that of any other commonly used cognitive battery used in stroke (Quinn et al., 2018). Combined with Bayesian predictive modeling, such a large sample allows correction for a broad range of demographic and neuropsychiatric variables, even when these are proportionally uneven between groups. For example, in our study, approximately 88% of the stroke group used a tablet to answer the questions, while only 20% of the control did so. English as a second language was present in 34% of patients, compared to 4% of controls. Nonetheless, these number at least 231 control individuals in absolute terms for each group, affording sufficient statistical power to draw reliable inferences regarding the impact of each independent variable. Notably, the sample size of this control sub-population is comparable to—or even larger than—the entire normative cohort of commonly used cognitive assessments in stroke (Demeyere et al., 2015; Quinn et al., 2018).

The evidence presented in this paper is further corroborated by recent studies from our group, showing the feasibility of online cognitive testing for identification and monitoring of impairments in neurological disorders, such as traumatic brain injury, Parkinson’s disease, and autoimmune limbic encephalitis (Bălăeţ et al., 2024; Del Giovane et al., 2023; Shibata et al., 2024). Collectively, these studies have shown that online assessments correlate well with standard clinical evaluations while exhibiting higher sensitivity, essentially providing a hyper-resolution on cognitive deficits relative to common scales. The current results not only align with these findings, but also provide much more extensive reliability and validity metrics, showcasing the (a) robustness of the IC3 assessment, (b) its superiority against standard clinical screens, and (c) its ability to predict functional outcomes after stroke. These results provide supporting evidence for integration of such platforms in clinical care pathways, providing additional insight into clinical decisions, for instance, on when to perform potentially costly imaging or in-person testing during the follow-up period of the disease and inform rehabilitation decisions.

Nevertheless, it is important to consider the current findings in the context of certain limitations. Similar to most standard pen-and-paper tests, the IC3 tasks were administered in a predetermined order. Consequently, missing data were more likely to occur for tasks toward the latter part of the battery, potentially leading to underrepresenting participants with higher level of impairments for these tasks. However, it is worth noting that this phenomenon is inherent in all cognitive assessment methodologies, and the inability to complete the assessment can itself be considered a meaningful outcome measure. In the current study, only patients who had completed at least 50% of the IC3 assessment were included in the analysis, with the last task of the IC3 containing 83/90 (92.2%) patients. For comparison 4,782/6,364 (75%) control respondents completed the last task, surpassing the normative sample size for most conventional assessment tools.

We showed that the IC3 has superior sensitivity to MoCA. Here, MoCA was used as the approximate ground truth for detecting post-stroke impairments, as this was routinely available as a clinical screening test in our patients. Although there is evidence for superiority of MOCA cognitive screening in patients with stroke compared to commonly used general cognitive screens (Dong et al., 2010), MoCA is likely to underestimate post stroke cognitive impairment (Chan et al., 2017; Demeyere et al., 2016). The optimal gold standard for defining clinically relevant impairment would require a detailed neuropsychological assessment that considers the patient’s pre-morbid level of cognitive functioning and the required cognitive demands faced by the patient. In the absence of such data in our patient cohort, and the likelihood that MoCA missed milder impairments, calculating specificity or false positive rate of IC3 against MoCA will be erroneous and misleading as noted previously (Roberts et al., 2024). In keeping with this, the IC3 specificity for overall cognitive domains against MOCA is relatively low (*M* = 0.45; range = 0.16–0.80%). Future work assessing the sensitivity and specificity of IC3 technology against more sensitive stroke-specific tests and in a larger sample size of patients is needed, and is currently underway. In addition, future work will be needed to address convergent validity of specific IC3 cognitive domains, in patient populations with specific cognitive impairments, beyond the validation presented in this paper using the global summary metric.

The patient data presented in this study was collected in hospital wards or in a clinical setting, via a semi-supervised approach. Specifically, the researcher was present in the room with the patient, but did not interfere or provide any suggestions to the patient on how to complete the tasks. Overall, we found that the assessment was well-tolerated and that patients engaged with it, in great part aided by the short format of the tasks, and the possibility of taking breaks after each task. More detailed information related to patient suitability for IC3, engagement and tolerance is compiled under Supplementary Table 1.

To maximize engagement of more severely impaired patients, the IC3 allows for unlimited number of breaks to be taken at the end of each task and provides built-in task/trial skipping functions to minimize fatigue in these patients. This was particularly relevant given that the average duration of task completion in patients was just under an hour. Nevertheless, these patients will not be suitable for unsupervised testing given their well-documented issues with engagement (Bill et al., 2013). The unsupervised administration is most likely feasible for patients with mild-to-moderate impairment, who have the highest potential for regaining independence and who may benefit most from personalized treatments and rehabilitation.

Furthermore, cognitive evaluations during the acute or chronic phases present different requirements and challenges. The problem of heterogeneity of cognitive impairments between patients and within individual patients at different stages of recovery is well established. While we did not note a difference in sensitivity of the IC3 with respect to stroke recovery phase (Figure 7B), future studies are needed to examine how well the digital technology can capture impairments at different recovery stages separately in larger samples. In addition, future studies could investigate whether the test–retest reliability observed in control participants also holds true for patients, whose performance typically shows greater variability within tasks and across time points. Such a detailed comparison was outside the scope of the current study.

Due to its cost-effectiveness and scalability, IC3 can be further developed for wide adoption as a clinical diagnostic and monitoring tool for patients with stroke and related vascular disorders. This will be tested in clinical trials within the health care setting. Such implementation will facilitate the detection and longitudinal monitoring of cognitive impairment after stroke at minimal cost. Given its sensitivity to mild impairment, IC3 can be used as the main cognitive outcome measure in clinical research studies in patients with stroke. We are currently adopting this approach in a longitudinal observational study of post-stroke cognition alongside blood biomarkers and brain imaging to identify mechanisms of recovery following stroke (Gruia et al., 2023). It is anticipated that these findings will inform tailored, personalized rehabilitation strategies for more effective recovery, a prospect not achievable with current assessment methodologies.

## Supplemental Material

sj-docx-1-asm-10.1177_10731911251381572 – Supplemental material for Development and Validation of the IC3: An Online Remote Assessment Technology for Deep Phenotyping and Monitoring of Cognitive Impairment After Stroke Supplemental material, sj-docx-1-asm-10.1177_10731911251381572 for Development and Validation of the IC3: An Online Remote Assessment Technology for Deep Phenotyping and Monitoring of Cognitive Impairment After Stroke by Dragos-Cristian Gruia, Valentina Giunchiglia, Aoife Coghlan, Sophie Brook, Soma Banerjee, Joseph Kwan, Peter J. Hellyer, Adam Hampshire and Fatemeh Geranmayeh in Assessment

## Data Availability

A subset of the datasets used can be made available on reasonable request from the corresponding author and upon institutional regulatory approval.
